# Impaired longitudinal deformation measured by speckle-tracking echocardiography in children with end-stage renal disease

**DOI:** 10.1007/s00467-016-3362-0

**Published:** 2016-05-17

**Authors:** Maike van Huis, Nikki J. Schoenmaker, Jaap W. Groothoff, Johanna H. van der Lee, Maria van Dyk, Marc Gewillig, Linda Koster, Ronald Tanke, Marc Lilien, Nico A. Blom, Luc Mertens, Irene M. Kuipers

**Affiliations:** Department of Pediatric Nephrology, Emma Children’s Hospital, Academic Medical Center (AMC) Amsterdam/University of Amsterdam, Amsterdam, The Netherlands; Pediatric Clinical Research Office, Emma Children’s Hospital, Academic Medical Center Amsterdam, Amsterdam, The Netherlands; Department of Pediatric Nephrology, University Hospital Leuven, Leuven, Belgium; Department of Pediatric Cardiology, University Hospital Leuven, Leuven, Belgium; Department of Pediatric Nephrology, University Medical Center (UMC) St. Radboud/Radboud University, Nijmegen, The Netherlands; Department of Pediatric Cardiology, UMC St. Radboud/Radboud University, Nijmegen, The Netherlands; Department of Pediatric Nephrology, Wilhelmina Children’s Hospital, UMC Utrecht/Utrecht University, Utrecht, The Netherlands; Department of Pediatric Cardiology, Emma Children’s Hospital, AMC Amsterdam/University of Amsterdam, Amsterdam, The Netherlands; Cardiology, The Hospital for Sick Children, The University of Toronto, Toronto, ON Canada

**Keywords:** Cardiovascular Imaging, Pediatric, Systolic dysfunction, Longitudinal strain, Children

## Abstract

**Background:**

Left ventricular dysfunction is an important co-morbidity of end-stage renal disease (ESRD) and is associated with a poor prognosis in the adult population. In pediatric ESRD, left ventricular function is generally well preserved, but limited information is available on early changes in myocardial function. The aim of this study was to investigate myocardial mechanics in pediatric patients with ESRD using speckle-tracking echocardiography (STE).

**Methods:**

Echocardiographic studies, including M-mode, tissue Doppler imaging (TDI) and STE, were performed in 19 children on dialysis, 17 transplant patients and 33 age-matched controls. Strain measurements were performed from the apical four-chamber and the short axis view, respectively.

**Results:**

The interventricular and left ventricular posterior wall thickness was significantly increased in dialysis and transplant patients compared to healthy controls. No significant differences were found in shortening fraction, ejection fraction and systolic tissue Doppler velocities. Dialysis and transplant patients had a decreased mean longitudinal strain compared to healthy controls, with a mean difference of 3.1 [95 % confidence interval (CI) 2.0–4.4] and 2.7 (95 % CI 1.2–4.2), respectively. No differences were found for radial and circumferential strain.

**Conclusions:**

Speckle-tracking echocardiography may reveal early myocardial dysfunction in the absence of systolic dysfunction measured by conventional ultrasound or TDI in children with ESRD.

## Introduction

Cardiovascular disease is highly prevalent in children and adults with end-stage renal disease (ESRD) and has been shown to be one of the main causes of mortality [1–4]. In young adults with ESRD, left ventricular hypertrophy (LVH) and impaired systolic function are found even at early stages of chronic kidney disease (CKD) [5–10]. In children with ESRD, systolic LV function generally seems to be well preserved, as described in observational studies using two-dimensional (2D) echocardiography and tissue Doppler measurements [7, 11].

Newer echocardiographic techniques, such as speckle-tracking echocardiography (STE), allow the study of myocardial deformation and myocardial mechanics [12–14]. STE has been used to describe early changes in myocardial mechanics prior to changes in the ejection fraction (EF). Studies in adults and children exposed to anthracyclines have shown that changes in longitudinal strain can be observed prior to changes in EF [15–17]. The same has been described in children with Duchenne cardiomyopathy [18]. In adults with CKD, a deterioration in renal function (estimated glomerular filtration rate) has been shown to be associated with a decline in strain values [19, 20], while in adults with ESRD, a decreased longitudinal strain has been shown to be a significant risk factor for all-cause mortality [21]. The aim of the study reported here was to identify early changes in myocardial mechanics in pediatric patients with ESRD using STE as the imaging modality.

## Methods

### Subjects

This is a multicenter prospective cohort study which recruited patients, aged 0–19 years, in three academic medical centers (AMC) in the Netherland (Emma Children’s Hospital AMC Amsterdam, Radboud University AMC and University Medical Center Utrecht) between 1 October 2007 and 1 April 2015. Children with a congenital heart disease were excluded. These three centers are involved in the Renal Insufficiency therapy in Children–Quality assessment and improvement (RICH-Q) project, in which all Dutch and Belgian centers providing pediatric renal replacement therapy (RRT) collaborate to improve the quality of care [22].

Controls were selected from a database of healthy Dutch children without any medical history who had been evaluated at the cardiology department of the respective AMC for a benign murmur, a positive family history of structural cardiac abnormalities or miscellaneous complaints that proved to be non-cardiac. The groups were matched for age. We assessed prevalence of hypertension in the patients, with hypertension defined as a blood pressure (BP) measurement of >95th percentile on at least three occasions based on gender, age and height according to the Fourth Report on the Diagnosis, Evaluation, and treatment of High Blood Pressure in Children and Adolescents [23], irrespective of use of antihypertensives. The body mass index* Z*-score was calculated based on gender and age according to the 2000 Centers for Disease Control and Prevention growth charts [24].

### Echocardiographic measurements

Echocardiographic assessment in patients undergoing hemodialysis (HD) was performed after a HD session.

All children were studied using the Vivid 7 ultrasound system (GE Medical Systems, GE Healthcare Life Sciences, Pittsburgh, PA) using a standardized protocol. Measurements of LV size and function were performed according to the guidelines published by the American Society of Echocardiography [25]. M-mode echocardiography was performed from the parasternal long axis views. Assessment of LV dimensions included: end-diastolic interventricular septum thickness (IVSd), LV end-diastolic and end-systolic diameter (LVEDd and LVED, respectively) and diastolic LV posterior wall thickness (LVPWd). The shortening fraction (SF) (%) was calculated. The LV mass index (LVMI) was calculated according to the Devereux formula, including a correction for height indexed to the power of 2.7 [26]. LVH was defined as an LVMI (g/m^2.7^) of >95th percentile according to the normal values for age and gender published by Khoury et al. [26]. Early and late mitral valve inflow velocities (E and A, respectively) were measured from the apical four-chamber view and the E/A ratio calculated. Each variable was measured three times, and the mean was calculated. Pulsed-wave tissue Doppler imaging (TDI) images were obtained from the apical four-chamber view. Tissue Doppler tracings were measured in the basal interventricular septum (IVS) and the basal LV lateral wall. Peak systolic (s′) and early diastolic (e′) velocities were measured in three consecutive cycles and averaged for both IVS (IVS s′) and basal LV wall (LV s′). Septal E/e′ and mitral E/e′ ratios were calculated. The ejection fraction (EF) was measured from the two-chamber and four-chamber views using the biplane Simpson’s method.

**Fig. 1:**
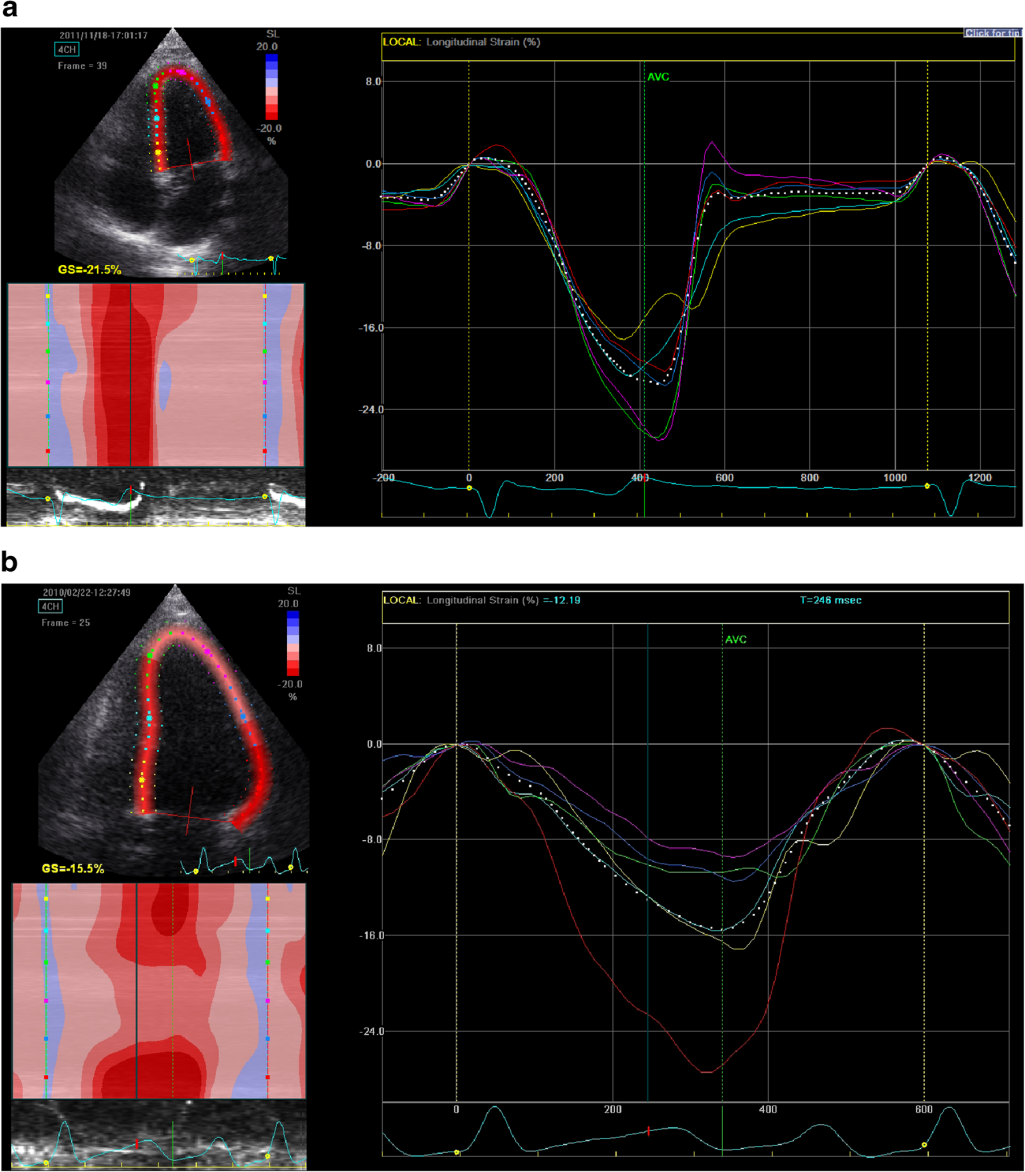
Two-dimensional speckle-tracking echocardiography (STE) for left ventricular (LV) longitudinal strain (LS) in a healthy control subject (**a**) and a child with end-stage renal disease (ESRD) (**b**). The mean LS (*white dotted line*) is the mean of the six segments of the myocardium of the left ventricle, (e.g.* yellow* basal septum,* light blue* mid septum,* green * apical septum,* red * basal lateral,* dark blue * mid lateral,* purple* apical lateral). The mean LS can be seen to be significantly lower in the child with ESRD (**b**) than in the healthy control subject (**a**)

2D grayscale images were acquired in the parasternal apical four-chamber view at a frame rate of between 70 and 90 frames per second [27]. Three consecutive cardiac cycles were acquired. Off-line analysis was performed using the EchoPac workstation (GE Medical Systems). Briefly, the endocardial border was manually traced at end systole (starting at mid-septum for the short axis and at the basal septum from the apical four-chamber view). Tracking was automatically performed, and the analysis was accepted after visual inspection and when the software indicated adequate tracking. If tracking was sub-optimal the endocardial border was retraced. Lagrangian radial ε and strain rate (SR) curves and circumferential ε curves from the short-axis view (6 segments: anterior septum, anterior, lateral, posterior, inferior and septum) and longitudinal ε curves from the apical four-chamber view (6 segments: basal septum, mid septum, apical septum, apical lateral, mid lateral and basal lateral) were obtained. The automated timing of aortic valve closure was used, and end-systolic strain values were measured. Mean longitudinal strain (LS), radial strain (RS) and circumferential strain (CS) were obtained by calculating the average strain values measured in each myocardial region (Fig. 1). LS is a negative value and thus represents shortening. A less negative, i.e. a ‘higher’ value, indicates less shortening, indicative of worse systolic LV function.

To assess the intra-observer variability of the STE measurements, the same observer—blinded to the groups—re-analyzed 25 echocardiograms, ten from ESRD patients and 15 from healthy controls, after a period of at least 2 weeks.

### Statistical analysis

All analyses were performed using SPSS ver. 22.0 for Windows statistical software (IBM Corp., Armonk, NY). Values are presented as mean ± standard deviation unless stated otherwise. An independent samples* t* test or Mann–Whitney test was used to compare the means of continuous variables when appropriate. Categorical values were compared using the chi-square test or Fisher exact test, where indicated. To assess intra-observer reproducibility, we calculated the coefficient of variation (CV) as the ratio of the standard deviation of the differences of the repeated measurements and the mean of all measurements in all individuals (grand mean). The CV gives an indication of the measurement error as a percentage of the mean value in the study population. Parameters related to systolic function were compared between the ESRD and the control group using multiple linear regression analysis to adjust for confounding. Potential confounders were age, gender and body surface area (BSA). If the regression coefficient of the central determinant ‘ESRD’ changed by >10 % after the addition of a particular variable to the regression model, this variable was considered to be a confounder and kept in the final model. Patients on dialysis and transplantation therapy were compared in order to assess the influence of RRT modality. Linear regression was used to analyze the association between total duration on RRT and echocardiographic measurements.

## Results

Measurements obtained in 36 children with ESRD and 33 healthy control subjects were analyzed. At the time of the echocardiography, 19 children were on dialysis (15 on HD and 4 on peritoneal dialysis) and 17 were transplant recipients. Of the 17 transplant recipients, 11 (65 %) had been on dialysis before this study. The median time on RRT at the time of the echocardiography was 1.4 (range 0.0–15.6) and 2.7 (range 0.1–4.8) years for the patients on dialysis and renal transplant recipients, respectively. The median time between transplantation and echocardiography was 13 (range 0.3–40) months. Eight (42 %) patients on dialysis and six patients (35 %) with a renal transplant were diagnosed with arterial hypertension. The characteristics of the children on dialysis, the transplant recipients and the healthy controls are shown in Table 1.

**Table 1:** General characteristics of the study population

General characteristics	Study population			*P* value		
	Dialysis (*n* = 19)	Transplant recipients (*n* = 17)	Healthy controls (*n* = 33)	Dialysis vs. healthy controls	Transplant recipients vs. healthy controls	Dialysis vs. transplant recipients
Male	12 (63 %)	12 (70 %)	16 (49 %)	0.78^a^	0.08^a^	0.45^a^
Hypertension	8 (42 %)	6 (35 %)	0 (0 %)	<0.001^a^	0.001^a^	0.74^a^
RRT duration (years)	1.4 [0.0–15.6]	2.7 [0.1–4.8]	-	-	-	0.30^b^
GFR (ml/min/1.73 m^2^)	7.9 [4.0–27.0]	60.3 [7.2–95.7]	-	-	-	-
Hemoglobin (mmol/l)	6.7 [5.3–8.0]	7.2 [5.4–8.5]	-	-	-	0.07
BSA (m^2^)	1.3 ± 0.4	1.3 ± 0.4	1.4 ± 0.4	0.67^b^	0.63^b^	0.85^b^
BMI* Z*-score	-0.1 [−3.1 to 1.6]	0.7 [−1.0 to 2.6]	0.2 [−3.8 to 1.7]	0.12^b^	0.21^b^	0.03^b^
Age (years)	15.1 [1.2–17.9]	13.7 [4.6–18.4]	12.5 [2.4–18.3]	0.24^b^	0.47^b^	0.89^b^

Values in table are presented as the median with the range in square brackets, a number (*n*) with the percentage in parenthesis or the mean ± standard deviation (SD)

*RRT* renal replacement therapy, *GFR* glomerular filtration rate (calculated according to the 2009 Schwartz formula), *BSA* body surface area (according to the Dubois and Dubois formula), *BMI* body mass index

^a^Fisher's exact test

^b^ Mann-Whitney U test

### Diastolic function

The E/A ratio did not differ significantly between the patients and healthy controls. TDI measurements were available in 16 children on dialysis, 15 transplant recipients and 27 healthy controls (Table 2). After adjustment for BSA, both children on dialysis and transplant recipients had significantly lower e′ values in the IVS and LV lateral basal segments, resulting in increased E/e′ ratios in both segments which did not, however, reach statistical significance.

**Table 2:** Tissue Doppler measurements

Characteristics	Study population			Dialysis vs. healthy controls^a^		Transplant recipient vs. healthy controls^a^		Dialysis vs. transplant recipient	
	Dialysis (*n* = 16)	Transplant recipients (*n* = 15)	Healthy controls (*n* = 27)						
				Mean difference [95 % CI]	*P* value	Mean difference [95 % CI]	*P* value	Mean difference [95 % CI]	*P* value
IVS S′	7.7 ± 1.7	7.8 ± 1.2	8.4 ± 1.0	0.5 [−0.4 to 1.5]	0.247	0.5 [−0.2 to 1.2]	0.16	0.1 [−1.0 to 1.3]	0.85
IVS E′	10.3 ± 2.8	11.0 ± 1.2	14.0 ± 2.0	3.7 [2.0–5.4]	<0.001	3.2 [1.8–4.4]	<0.001	0.6 [−1.1 to 2.8]	0.47
LV S′	8.1 ± 2.6	8.5 ± 1.8	9.2 ± 2.8	1.1 [-0.8 to 3.0]	0.26	0.9 [−0.9 to 2.6]	0.32	0.4 [−1.3 to 2.2]	0.61
LV E′	13.7 ± 4.8	15.9 ± 3.0	19.3 ± 3.2	5.5 [2.8–8.2]	<0.001	3.4 [1.2–5.5]	0.01	1.9 [−1.2 to 5.0]	0.21
	Dialysis (*n* = 15)	Transplant recipients (*n* = 13)	Healthy controls (*n* = 23)						
Septal E/E′ ratio	8.9 ± 3.7	8.3 ± 2.7	6.8 ± 2.6	1.5 [−0.8 to 3.8]	0.20	1.1 [−0.7 to 2.9]	0.24	0.4 [−1.9 to 2.5]	0.72
Mitral E/E′ ratio	6.6 ± 3.0	5.8 ± 2.4	5.0 ± 2.0	1.0 [−0.7 to 2.7]	0.23	0.3 [−1.1 to 1.6]	0.69	0.5 [−1.3 to 2.5]	0.55

Data are presented as the mean ± SD, unless indicated otherwise

*CI* confidence interval, *IVS S*′ interventricular septum peak systolic velocity, *IVS E*′, interventricular septum early diastolic velocity, *LV S*′ left ventricular (LV) wall peak systolic velocity, *LV E*′ LV early diastolic velocity

^a^Groups were compared using multiple linear regression analysis to adjust for confounding by BSA

### Systolic function

Systolic function in the patients, as measured with TDI (IVS s′ and LVS s′), did not differ significantly from that of the healthy controls (Table 2).

Table 3 summarizes the results of the LV dimensions, LV SF and LV mass. There was no difference in SF or EF between the patients and controls. After adjustment for BSA and age, there was a significant effect of dialysis and transplant status on LVMI, which was increased in children on dialysis and transplanted children compared to healthy controls. LVH was diagnosed in 4 (21 %) of the dialysis children and 4 (24 %) of the transplant recipients.

**Table 3:** Conventional echocardiographic measurements

Characteristics	Study population			Dialysis vs. healthy controls^a^		Transplant recipient vs. healthy control^a^s		Dialysis vs. Transplant recipient^a^	
	Dialysis (*n* = 19)	Transplant recipients (*n* = 17)	Healthy controls (*n* = 33)						
				Mean difference [95 % CI]	*P* value	Mean difference [95 % CI]	*P* value	Mean difference [95 % CI]	*P* value
IVSd (mm)	6.9 ± 1.7	6.9 ± 1.0	5.9 ± 1.6	1.0 [0.2–1.8]	0.02	1.1 [0.4–1.7]	0.01	0.1 [−0.7 to 0.8]	0.89
LVEDd (mm)	43.3 ± 7.8	44.5 ± 5.1	43.8 ± 10	0.2 [−4.1 to 4.5]	0.93	1.6 [−2.2 to 5.5]	0.40	1.1 [−1.6 to 3.7]	0.41
LVPWd (mm)	7.0 ± 1.8	6.9 ± 1.4	5.8 ± 1.5	1.1 [0.3–1.9]	0.01	1.0 [0.2–1.7]	0.01	-0.1 [−1.0 to 0.8]	0.86
LVMI (g/m^2.7^)	36.3 ± 12.7	39.5 ± 11.8	28.5 ± 9.9	7.9 [0.9–14.9]	0.03	10.4 [4.3–16.6]	0.001	3.2 [−4.2 to 10.8]	0.38
LVH	4 (21 %)	4 (24 %)	0 (0 %)	-	0.01^b, c^	-	0.01^b, c^	-	0.59^b, c^
SF (%)	36.2 ± 4.9	40.1 ± 4.4	37.2 ± 7.9	0.3 [−4.3 to 5.0]	0.89	3.3 [−0.9 to 7.7]	0.12	3.7 [0.6−6.8]	0.02
	Dialysis (*n* = 12)	Transplant recipients (*n *= 10)	Healthy controls (*n* = 8)						
EF 4CH (%)	50.9 ± 7.7	55.0 ± 5.0	55.5 ± 5.8	0.3 [−8.1 to 8.6]	0.95	1.1 [−5.6 to 7.9]	0.72	3.2 [−3.5 to 9.9]	0.33
	Dialysis (*n* = 14)	Transplant recipients (*n *= 14)	Healthy controls (*n* = 25)						
MV E	84.0 ± 30.6	89.0 ± 28.0	87.8 ± 35.8	8.8 [−16.8 to 34.5]	0.49	4.0 [−18.8 to 26.9]	0.72	6.3 [−15.5 to 28.1]	0.56
MV a	46.5 ± 20.9	54.0 ± 9.0	44.5 ± 16.8	2.2 [−11.2 to 15.7]	0.73	9.7 [−0.9 to 20.4]	0.07	8.2 [−4.9 to 21.6]	0.21
MV E/a ratio	2.0 ± 0.8	2.9 ± 3.7	2.1 ± 0.5	0.2 [−0.3 to 0.6]	0.47	0.7 [−0.8 to 2.2]	0.32	0.8 [−1.2 to 2.8]	0.42

Data are presented as mean ± SD or as a number (*n*) with the percentage in parenthesis, unless indicated otherwise

*IVSd* interventricular septal thickness in diastole, *LVEDd* LV end-diastolic diameter, *LVPWd* LV posterior wall thickness in diastole, *LVMI* LV mass index, *LVH* LV hypertrophy, *SF* shortening fraction, *EF 4CH* ejection fraction, 4-chamber, *MV E* early filling velocity, *MV* a, late filling velocity, *CI* confidence interval

^a^All groups were compared using multiple linear regression analysis to adjust for confounding by body surface area (BSA)

^b^Fisher's exact test

^c^No adjustment for BSA

### Speckle-tracking echocardiography

There were no significant differences between the repeated measurements: the CV of LS was 3 %, and the mean difference between measurements was 0.2 [95 % confidence interval (CI) −1.5 to 1.9]. Table 4 summarizes the longitudinal, radial and circumferential strain measurements. The mean LS was significantly reduced in patients on dialysis and after renal transplant when compared with the controls, with a mean difference between the dialysis and transplantation groups versus controls of 3.1 [95 % CI 2.0–4.4] and 2.7 [95 % CI 1.2–4.2] respectively (both* P* <0.001). The RS and CS measurements did not differ significantly between the patient groups and the controls, with a mean difference between the RS in the dialysis and transplantation groups versus controls of 0.5 [95 % CI −8.2 to 7.1] and 5.0 [95 % CI −2.6 to12.6], respectively. The mean difference between the CS in the dialysis and transplantation groups versus the controls was 0.5 [95 % −1.7 to 2.8] and 0.5 [95 % −1.9 to 2.9], respectively. LS decreased with 0.05 per unit increase of LVMI, but it did not differ between patients with and patients without LVH (mean difference of −1.0 [−3.4 to 1.5].

**Table 4:** Speckle Tracking measurements

	Dialysis	Transplant recipients	Healthy controls	Dialysis vs. healthy controls^a^		Transplant recipients vs. healthy controls^a^		Dialysis vs. transplant recipients^a^	
Characteristics				Mean difference [95 % CI]	*P* value	Mean difference [95 % CI]	*P* value	Mean difference [95 % CI]	*P* value
Longitudinal strain (%)	*n* = 19	*n* = 17	*n* = 33						
Basal septum	−15.2 ± 3.4	−16.8 ± 2.6	−15.4 ± 2.5						
Mid septum	−18.9 ± 2.4	−19.5 ± 1.9	−20.1 ± 1.8						
Apical septum	−20.2 ± 5.3	−19.6 ± 4.5	−23.0 ± 4.0						
Basal lateral	−17.0 ± 5.0	−15.2 ± 5.4	−18.8 ± 5.6						
Mid lateral	−15.0 ± 5.4	−15.2 ± 6.0	−19.8 ± 3.3						
Apical lateral	−17.0 ± 5.1	−16.8 ± 7.5	20.6 ± 4.9						
Mean Longitudinal strain	−16.6 ± 2.8	−16.7 ± 3.4	−19.4 ± 2.1	3.1 [2.0–4.4]	<0.001	2.7 [1.2–4.2]	0.001	0.4 [−1.7 to 2.5]	0.68
Radial strain (%)	*n* = 16	*n* = 17	*n* = 33						
Basal septum	18.0 ± 9.8	25.1 ± 14.6	22.6 ± 10.7						
Mid septum	19.8 ± 13.9	20.4 ± 13.2	17.9 ± 15.7						
Apical septum	27.1 ± 21.0	26.9 ± 16.7	19.1 ± 19.5						
Basal lateral	14.6 ± 12.1	21.3 ± 21.7	17.9 ± 10.5						
Mid lateral	28.5 ± 21.2	29.6 ± 18.8	24.1 ± 13.8						
Apical lateral	37.5 ± 29.3	35.7 ± 18.1	30.6 ± 23.8						
Mean radial strain	24.3 (14.5)	26.5 ± 13.3	22.0 ± 12	0.5 [−8.2 to 7.1]	0.88	5.0 [−2.6 to 12.6]	0.19	4.6 [−4.5 to 13.7]	0.31
Circumferential strain (%)	*n *= 16	*n* = 15	*n* = 33						
Basal septum	21.0 ± 6.8	24.2 ± 5.3	21.2 ± 5.2						
Mid septum	15.7 ± 5.9	21.5 ± 7.3	17.5 ± 6.0						
Apical septum	13.7 ± 6.0	15.5 ± 5.4	15.5 ± 4.9						
Basal lateral	21.3 ± 5.0	21.2 ± 4.4	21.8 ± 3.9						
Mid lateral	17.0 ± 5.9	16.2 ± 5.0	16.8 ± 6.3						
Apical lateral	15.2 ± 7.7	12.5 ± 6.1	14.5 ± 6.5						
Mean circumferential strain	17.3 ± 3.4	17.9 ± 4.1	17.4 ± 4.0	0.5 [−1.7 to 2.8]	0.65	0.5 [−1.9 to 2.9]	0.66	1.0 [−1.7 to 3.6]	0.45

Data in table are presented as the mean ± SD, unless indicated otherwise

*CI* confidence interval

^a^All groups were compared using multiple linear regression analysis to adjust for confounding by body surface area (BSA)

### Influence of RRT and presence of hypertension

There were no significant differences between the renal transplant and the dialysis groups with respect to conventional echocardiographic measurements, TDI or STE measurements (Tables 2, 3, 4). No significant association was found between total duration of RRT and the echocardiographic parameters. LS and LVMI did not differ between patients with and without hypertension. The mean difference in LS and LVMI for patients with and without hypertension was 1.1 [95 % CI −2.4 to 1.9] and 4.2 [−7.3 to 9.9], respectively.

## Discussion

Our study demonstrates that in pediatric patients with ESRD, longitudinal LV strain was significantly lower in both dialysis and renal graft recipients compared with normal controls, while radial and circumferential function were not different. Also, SF and EF were generally normal. These findings suggest that in children with ESRD, LV systolic performance assessed by EF is generally normal, whereas STE detects changes in longitudinal deformation and diastolic function.

In hypertensive patients, diastolic dysfunction precedes systolic function [28], and this has been described previously in pediatric ESRD [10, 29, 30]. We found decreased e′-velocities, suggestive of reduced early relaxation and increased E/e′ ratio which could indicate higher filling pressures in the patients. The increased filling pressures could be related to volume status or represent reduced LV compliance and may be caused by uremic toxins inducing an inflammatory response or be related to the maladaptive hypertrophic response in this patient population group [31]. Thse hypotheses need further study by cardiac magnetic resonance imaging or other imaging modalities.

In ESRD, hypertension and uremic factors are independently associated with both LVH and ventricular dysfunction [32]. Hypertension in non-uremic patients can either lead to concentric hypertrophy with normal or even increased EF in early stages or to eccentric hypertrophy [33]. Apparent systolic dysfunction occurs only in advanced stages of hypertension-induced LVH. In adult ESRD patients, however, systolic dysfunction may occur at a relative early stage, most likely as a result of myocardial fibrosis induced by chronic inflammation or in direct response to uremic toxins. This fibrosis is an important trigger of electric myocardial instability and hence arrhythmia [32]. In addition, endothelial dysfunction, another hallmark of ESRD, may lead to an inadequate vasodilatory response in the thickened left ventricle and subsequently local ischemia, hereby further enhancing fibrosis [34]. Consequently, the absence of systolic dysfunction based on EF assessment may result in an underestimation of the existence of important systolic myocardial changes by the uremic milieu.

In our patients, although there was an increased LVMI and hence ventricular hypertrophy, no systolic dysfunction was found with conventional echocardiography (normal SF and EF) and with TDI (IVS s′ and LVS s′). Nevertheless, STE showed a decreased LS in our patients, suggesting that longitudinal function is reduced in the patient group, while radial and circumferential function is preserved. LV concentric hypertrophy is mainly caused by hypertrophic response in the mid-myocardial layers, which are mainly more circumferentially oriented. This compensates for the reduction in longitudinal function and can explain the preserved EF. Changes in longitudinal function with preserved EF have been described in other disease populations, mainly in patients with LVH [35]. Hothi et al. [36] described a decreased LS in children on dialysis with preservation of global function.

Our findings are consistent with data obtained in hypertensive adults as reported by Imbalzano et al. [37]. In patients with hypertension, the changes are most prominent in the basal part of the IVS. A decrease in longitudinal function precedes changes in circumferential and radial function in patients with LVH due to pressure overload, whereas in hypertrophic cardiomyopathy or systemic disease, not only longitudinal, but also radial strain can be impaired [35]. In our study, LV mass increased in 21 % of the dialysis patients and 24 % of the renal transplant patients, suggesting some degree of LVH, but the presence of LVH did not seem to be a risk factor for the decrease in LS, in turn suggesting that the changes in strain can be present in ESRD patients in the absence of LVH. However, it should be noted that the measurement of LVH in pediatric ESRD has already been shown to be less reliable, as demonstrated by Schoenmaker et al. [38].

In ESRD, volume overload and myocardial ischemia induced by HD can cause mechanical dyssynchrony by imbalances in the stretching and shortening of myocardial fibers, which results in a pathological STE pattern that may affect systolic function [39, 40]. Cardiac fibrosis is highly prevalent in patients with ESRD, but the origin and mechanisms of fibrosis in the heart are have not yet been completely elucidated [41–43]. In addition, in patients with CKD, myocardial dysfunction is not merely the result of cardiac remodeling; especially in dialysis patients, impaired myocardial reserve may induce recurrent myocardial stunning and hence myocardial dysfunction. In ESRD, high serum phosphate levels, high fibroblast growth factor 23 (FGF23) levels and low serum Klotho levels are considered to play a role in cardiac hypertrophy and cardiac fibrosis. High serum FGF23 levels are primarily associated with LVH, whereas low serum Klotho levels and hyperphosphatemia are associated with endothelial dysfunction, atherosclerosis and fibrosis [41, 44]. It is possible that high serum phosphate levels and low serum Klotho levels may independently of high FGF23 levels induce cardiac fibrosis, resulting in dyssynchronous myocardial function. However, this hypothesis is not supported by our data.

As previously described, changes in longitudinal myocardial function with preserved EF have been described in other disease populations, as well as in adult ESRD patients [15, 16, 20, 45–47]. In a recent systematic review and meta-analysis in adults (mean age >60 years), global LS was considered to have a superior prognostic value to EF for predicting major adverse cardiac events [45]. Furthermore, LS has been shown to be significantly associated with all-cause mortality in adults with chronic ischemic cardiomyopathy [hazard ratio 1.69 (95 % CI 1.33–2.15) per 5 % increase;* p* <0.001] [48]. Whether a reduction in LS can be used as a specific predictor of cardiovascular morbidity and mortality in children with ESRD remains to be established. This will require longitudinal follow-up studies in children with ESRD.

We found no significant differences in echocardiography measurements between children on dialysis and those on a functioning graft, nor did we find significant associations between duration on RRT and echocardiographic measurements. This suggests that duration of dialysis does not influence cardiac performance. However, we were unable to draw conclusions from our analysis of the association between dialysis vintage and cardiac phenotype as our study population was too heterogeneous.

### Limitations

The major limitation of this study is the small sample size. The study was limited to the three hospitals in the RICH-Q project where the same ultrasound equipment was used, as we wanted to avoid the effect of inter-machine variability on the measurements. Machines of different vendors produce different values for speckle-tracking strain-derived parameters [49] and, consequently, comparison is more difficult. Despite adopting a prospective protocol, we were unable to acquire all data prospectively from all patients because some images were of poor quality, limiting the number of available scans. Adjustment of echocardiographic measurements to body size in our population was challenging as many ESRD patients had a shorter height and smaller BSA compared to age-matched controls. We were not able to assess inter-observer variability; however a recent study has shown a good reproducibility of strain measurements with low inter- and intra-observer relative mean errors, with lower errors than for EF and most other conventional echocardiographic parameters [50].

## Conclusion

We found a decreased LS, suggestive of systolic dysfunction in pediatric ESRD patients, while systolic function measured both by conventional echocardiography and TDI was preserved. STE may reveal early myocardial dysfunction in the absence of systolic dysfunction in ESRD children. The long-term importance of these findings warrants further investigation and follow-up.
